# Targeting fatty acid synthase modulates sensitivity of hepatocellular carcinoma to sorafenib via ferroptosis

**DOI:** 10.1186/s13046-022-02567-z

**Published:** 2023-01-06

**Authors:** Yan Li, Wenjuan Yang, Yuanyuan Zheng, Weiqi Dai, Jie Ji, Liwei Wu, Ziqi Cheng, Jie Zhang, Jingjing Li, Xuanfu Xu, Jianye Wu, Mingwei Yang, Jiao Feng, Chuanyong Guo

**Affiliations:** 1 Department of Gastroenterology, Shanghai Tenth People’s Hospital, Tongji University School of Medicine, Shanghai, 200072 China; 2 Department of Emergency, Shanghai Tenth People’s Hospital, Tongji University School of Medicine, Shanghai, 200072 China; 3 Department of Gastroenterology, Shidong Hospital, Shanghai, 200433 China; 4 Department of Gastroenterology, Putuo People’s Hospital, Tongji University, Shanghai, 200060 China; 5 Department of Oncology Radiotherapy, The First Affiliated Hospital of Anhui Medical University, Hefei, 230031 China

## Abstract

**Background:**

Sorafenib resistance is a key impediment to successful treatment of patients with advanced hepatocellular carcinoma (HCC) and recent studies have reported reversal of drug resistance by targeting ferroptosis. The present study aimed to explore the association of fatty acid synthase (FASN) with sorafenib resistance via regulation of ferroptosis and provide a novel treatment strategy to overcome the sorafenib resistance of HCC patients.

**Methods:**

Intracellular levels of lipid peroxides, glutathione, malondialdehyde, and Fe^2+^ were measured as indicators of ferroptosis status. Biological information analyses, immunofluorescence assays, western blot assays, and co-immunoprecipitation analyses were conducted to elucidate the functions of FASN in HCC. Both in vitro and in vivo studies were conducted to examine the antitumor effects of the combination of orlistat and sorafenib and CalcuSyn software was used to calculate the combination index.

**Results:**

Solute carrier family 7 member 11 (SLC7A11) was found to play an important role in mediating sorafenib resistance. The up-regulation of FASN antagonize of SLC7A11-mediated ferroptosis and thereby promoted sorafenib resistance. Mechanistically, FASN enhanced sorafenib-induced ferroptosis resistance by binding to hypoxia-inducible factor 1-alpha (HIF1α), promoting HIF1α nuclear translocation, inhibiting ubiquitination and proteasomal degradation of HIF1α, and subsequently enhancing transcription of SLC7A11. Orlistat, an inhibitor of FASN, with sorafenib had significant synergistic antitumor effects and reversed sorafenib resistance both in vitro and in vivo.

**Conclusion:**

Targeting the FASN/HIF1α/SLC7A11 pathway resensitized HCC cells to sorafenib. The combination of orlistat and sorafenib had superior synergistic antitumor effects in sorafenib-resistant HCC cells.

**Supplementary Information:**

The online version contains supplementary material available at 10.1186/s13046-022-02567-z.

## Introduction

Hepatocellular carcinoma (HCC) is the third leading cause of cancer-related death worldwide [1]. Because of the insidious symptoms, a high proportion of patients are initially diagnosed with an advanced stage. For the treatment of advanced HCC patients, sorafenib is the first and consistently certified first-line drug [2]. Sorafenib was reported as a multi-targeted tyrosine kinase inhibitor (TKI) that inhibited multiple intracellular kinases (RAF, wild-type and mutant BRAF) and cell surface kinases (KIT, FLT3, RET, VEGFR1-3 and PDGFRB). In addition, sorafenib may also antagonize HCC through other mechanisms, such as facilitating cellular scorching of macrophages, enhancing natural killer cells-triggered anti-HCC activity, and inducing cellular ferroptosis in HCC [3].

Ferroptosis is an iron dependent regulated cell death form characterized by unrestricted lipid peroxidation and subsequent plasma membrane damage [4]. Previous studies reported that sorafenib was a ferroptosis-specific activator targeting Solute carrier family 7 member 11 (SLC7A11) (a key subunit of the amino acid transport system x_c_^−^) [5, 6]. System x_c_^−^ imports cystine and outputs glutamate to support the synthesis of glutathione (GSH), an antioxidant tripeptide. GSH is a cofactor required for glutathione peroxidase 4 (GPX4) and is involved in the reduction of oxidized phospholipids (PLs) (PL-OOH) to the corresponding alcohols (PL-OH). Thus, inhibition of SLC7A11 by sorafenib can lead to GSH depletion and subsequent GPX4 inactivation, resulting in excessive accumulation of lipid peroxide in the cell membrane, leading eventually to ferroptosis.

Sorafenib sensitivity has also recently been shown to be positively associated with ferroptosis activity [5, 6]. Conceptually, ferroptosis is a metabolic form of cell death induced by oxidative stress [7]. Compared to normal cells, resistant tumor cells tend to have higher levels of antioxidant substances (such as GSH) to neutralize intracellular excessive reactive oxygen species (ROS), which in turn mitigates sorafenib-induced ferroptosis in HCC [8, 9]. Correspondingly, GSH depletion, GPX4 downregulation and lipid peroxide accumulation promoting ferroptosis results in drug-resistant HCC cells with impaired ability to resist oxidative damage, thereby enhancing their sensitivity to sorafenib [10, 11]. Thus, triggering ferroptosis is recognized as a valuable target for resensitizing HCC cells to sorafenib.

Since ferroptosis is caused by peroxidation of polyunsaturated fatty acids (PUFAs), regulation of lipid metabolism is a key determinant of susceptibility to ferroptosis. Theoretically, acetyl coenzyme A is carboxylated by acetyl coenzyme to form malonyl coenzyme A. Malonyl coenzyme A is then further converted by fatty acid synthase (FASN) to palmitic acid, a saturated fatty acid (SFA). Palmitoyl CoA or stearoyl CoA can subsequently be desaturated by stearoyl CoA desaturase-1 (SCD1) to form palmitoleic acid and oleic acid, respectively, both of which are monounsaturated fatty acids (MUFA) [12]. Upregulation of FASN can increase the synthesis of SFA and MUFA, and high saturated levels of membrane lipids protect cancer cells from ROS-induced damage [13], thus preventing and repairing sorafenib induced lipid peroxidation and responsible for sorafenib resistance. However, inhibition of FASN can induce enrichment of PUFAs, which is more susceptible to lipid peroxidation due to its multiple double bonds, leading to ferroptosis [13].

Moreover, FASN is significantly upregulated in cancers of the breast, ovary, and pancreas, especially when accompanied by treatment-resistant features [14–16]. However, relatively few studies have investigated the role of FASN in modulation of sorafenib resistance and the specific underlying mechanism remains unclear. In consideration of the crosstalk between ferroptosis and lipid metabolism [17], we speculated that targeting of FASN could reverse sorafenib resistance by modulating susceptibility to ferroptosis.

In our present study, we reported an important role for FASN in regulating sorafenib resistance in HCC cells. The results showed that blocking FASN expression promoted sorafenib-induced ferroptosis via downregulation of SLC7A11 expression, which reversed treatment resistance of HCC cells. Notably, we also found that orlistat, an inhibitor of FASN [18], enhanced the sensitivity of resistant cells to sorafenib both in vivo and in vitro. Our work therefore provided a new therapeutic target and strategy for sorafenib resistance in patients with advanced HCC.

## Materials and methods

### Cell lines and cell culture

Cultured Huh7, SMMC-7721, and 293T cells were purchased from the Cell Bank of Type Culture Collection (Chinese Academy of Sciences, Beijing, China). SMMC-7721 and Huh7 cells were maintained in Roswell Park Memorial Institute (RPMI) 1640 medium (Sigma-Aldrich Corporation, St. Louis, MO, USA) supplemented with 10% fetal bovine serum (FBS; Gibco, Carlsbad, CA, USA), while 293T cells were maintained in high-glucose Dulbecco’s Modified Eagle’s Medium (Sigma-Aldrich) supplemented with 10% FBS. All cell lines were cultured in an incubator at 37 °C under an atmosphere of 5% CO_2_/95% air. Short tandem repeats of four liver-specific genes in SMMC-7721 cells and the cycle threshold values obtained by quantitative real-time polymerase chain reaction (qRT-PCR) are provided in the supplemental materials. Sorafenib-resistant (SR) cell lines were cultured in accordance with standard methods, as previously described [19]. The resultant Huh7SR and 7721SR cells were continuously cultured in RPMI 1640 complete medium containing 2 µg/mL of sorafenib.

### Drugs and chemicals

Sorafenib and orlistat were purchased from MedChemExpress (catalog nos. HY-10,201 and HY-B0218, respectively; Monmouth Junction, NJ, USA). The following inhibitors and activators (all from Shandong Topscience Biotech Co., Ltd., Rizhao, China) were used in this study: erastin (T1765) at 10 µM, RSL3 (T3646) at 1 µM, ferrostatin-1 (T6500) at 10 µM, Z-VAD-FMK (carbobenzoxy-valyl-alanyl-aspartyl-[O-methyl]- fluoromethylketone) (T6013) at 10 µM, belnacasan (T6090) at 10 µM, necrostatin-1 (T1847) at 0.5 µM, and YC-1 (T4381) at 10 µM. All required co-incubation with the indicated cells for 24 h before sorafenib treatment. Hypoxic cells were treated with 250 µM CoCl_2_ (Selleck Chemicals, Houston, TX, USA) for 12 h.

### Antibodies

Antibodies against the following proteins were used for western blot analyses: SLC7A11 (ab175186 and ab216876; Abcam, Cambridge, MA, USA), hypoxia-inducible factor 1-alpha (HIF1α; 66730-1-Ig; Proteintech, Rosemont, IL, USA), FASN (A0461; ABclonal Technology, Woburn, MA, USA), and β-actin (AC026; ABclonal Technology).

### Cell transfection

Human full-length FASN, HIF1α, and SLC7A11 were used for overexpression studies with empty plasmids as controls. Small interfering RNA (siRNA) was used to knockdown expression of FASN. For short-term knockdown or overexpression, HCC and HCC-SR cells were transfected with siRNA or plasmids using Lipofectamine™ 3000 transfection reagent (Thermo Fisher Scientific, Waltham, MA, USA) in accordance with the manufacturer’s instructions.

### Cell-counting kit-8 (CCK-8) assay

The specified cells were seeded in wells of 96-well plates at approximately 2000 cells/100 µL per well and monitored for 5 days or at approximately 5000 cells/100 µL per well, then incubated with a specific drug for 24 h. Afterward, 10 µL of CCK-8 reagent (CK04; Dojindo Laboratories Co., Ltd., Kumamoto, Japan) were added to each well and the cells were incubated for an additional 2 h. Finally, the optical density at 450 nm was measured using a microplate reader.

### GSH and malondialdehyde (MDA) assays

The relative concentration of MDA in at least 1 × 10^7^ cells was measured using the MDA assay kit (M496; Dojindo Laboratories), while the concentration of GSH was measured using the GSSG assay kit (S0053; Beyotime Institute of Biotechnology, Shanghai, China) in accordance with the manufacturers’ instructions.

### Lipid peroxidation measurement

Lipid peroxidation were assessed using the fluorescent probe C11-BODIPY (581/591) (GC40165; GlpBio Technology, Montclair, CA, USA) according to the manufacturer’s protocol. Briefly, cells were treated as indicated, then collected and incubated with C11-BODIPY (581/591) at a final concentration of 2.5 µM for 30 min. The cells were then washed twice with phosphate-buffered saline (PBS) to remove residual C11-BODIPY. Lipid ROS of the cell suspensions were measured with a flow cytometer (LSR II; BD Biosciences, San Jose, CA, USA).

### Human specimens and immunohistochemical (IHC) staining

Tissue microarrays of human HCC tumor tissues and adjacent tissues were obtained from the First Affiliated Hospital of Anhui Medical University under institutional approvals. Paraffin-embedded tissue samples were cut into 4 μm-thick slices, which were dewaxed and dehydrated. After antigen repair, the slices were incubated with antibodies against SLC7A11 (dilution, 1:500; TD12509; Abmart, Shanghai, China), HIF1α (dilution, 1:100; 66730-1-Ig; Proteintech), FASN (dilution, 1:100; A0461; ABclonal Technology), and Ki-67 (dilution, 1:500; GB11141; Wuhan Servicebio Technology, Wuhan, China), followed by an anti-goat secondary antibody (Wuhan Servicebio Technology). Subsequently, the tissue slices were stained with 3,3′-diaminobenzidine and hematoxylin. Images were acquired under an optical microscope.

### RNA extraction and RT-qPCR

Total RNA was isolated from cells using TRIzol reagent (Invitrogen Corporation, Carlsbad, CA, USA) in accordance with the manufacturer’s instructions and then reverse transcribed into complementary DNA using the PrimeScript™ RT kit (Takara Bio, Shiga, Japan) with Hieff® qPCR SYBR Green Master Mix (Low Rox Plus) (11202ES03; Shanghai Yeasen Biotechnology, Shanghai, China), the primers listed in Table S1, and the QuantStudio Dx PCR system (Thermo Fisher Scientific). Relative mRNA expression was calculated using the 2^−ΔΔCt^ method and normalized against expression of β-actin as an internal control.

### Dual luciferase reporter gene assay

The CDS region of SLC7A11 was searched by NCBI, and nucleotides containing the SLC7A11 promoter (-2000 to 100 of the human SLC7A11 locus) were used to predict potential transcription factor binding sites. Potential binding sites for HIF1α to the SLC7A11 promoter region were predicted from the JASPAR database (https://jaspar.genereg.net/). The entire nucleic acid sequence of the promoter of SLC7A11 was used as the wild-type (WT). Nucleotides containing the WT SLC7A11 promoter fragment or binding site truncated mutant (MUT) SLC7A11 (SLC7A11-WT, SLC7A11-MUT1, and SLC7A11-MUT2) were cloned into pGL4.10 vector by IBSBIO (Shanghai, China). HEK 293T cells were seeded into 24-well plates and then cotransfected with HIF1α plasmid, pRL TK plasmid (renilla luciferase reporter vector), and pGL4.10 vector plasmids (or SLC7A11-WT, SLC7A11-MUT1, SLC7A11-MUT2 plasmids). After 24 h, the reporter gene cell lysate was added and the luciferase and renilla luciferase activities were measured using a dual luciferase reporter gene assay (RG027; Beyotime Institute of Biotechnology).

### Western blot and co-immunoprecipitation (Co-IP) analyses

Treated cells were lysed with radioimmunoprecipitation assay buffer (P0013B; Beyotime Institute of Biotechnology) supplemented with protease and phosphatase inhibitors on ice for 30 min and centrifuged at 12,000 rpm for 15 min at 4 °C. The total protein content was quantified using a bicinchoninic acid assay kit (20201ES76; Shanghai Yeasen Biotechnology) and equivalent amounts of proteins were separated by electrophoresis and then transferred using transfer buffer (WB4600; NCM Biotech, Suzhou, China) to polyvinylidene fluoride membranes, which were blocked with 5% milk in Tris-buffered saline with Tween® 20 detergent (TBST) and then incubated with primary antibodies at 4 °C overnight followed by appropriate secondary antibodies at room temperature for 1 h. Immunoreactive protein bands were visualized with an enhanced chemiluminescence system (Amersham™ Imager 600; GE Healthcare Bio-Sciences Corp., Piscataway, NJ, USA) and an Odyssey® M Infrared Imaging System ( LI-COR Biosciences, Lincoln, NE, USA).

Interactions between FASN and HIF1α were detected using the Pierce™ Classic Magnetic IP/Co-IP Kit (Thermo Fisher Scientific). In brief, cells were lysed on ice with IP lysis buffer containing protease and phosphorylation inhibitors and centrifuged at 12,000 × *g* for 10 min at 4 °C. After discarding the supernatant, the pelleted cells were incubated with antibody-coupled beads, washed three times with TBST, and then boiled in loading buffer containing sodium dodecyl sulfate for western blot analysis.

### Detection of intracellular Fe^2+^

Cells were inoculated in confocal dishes and incubated with 1 µM FerroOrange (F374; Dojindo Laboratories Co., Ltd.) working solution at 37 °C for 30 min. Then, intracellular Fe^2+^ was observed under a confocal microscope (LSM 780; Carl Zeiss AG, Jena, Germany).

### Immunofluorescence (IF) assay

Treated cells were inoculated on fibronectin-coated glass coverslips in 24-well culture plates. After attachment, the cells were fixed with 4% paraformaldehyde for 15 min, permeabilized with 0.3% Triton X-100 for 10 min, washed twice with phosphate-buffered saline, and fixed with 10% goat serum for 1 h. Afterward, the cells were incubated with primary antibodies at 4 °C overnight followed by the appropriate secondary antibodies at room temperature for 1 h. Meanwhile, the nuclei were stained with 4′,6-diamidino-2-phenylindole for 15 min. Finally, the cells were imaged under a confocal microscope (LSM 780; Carl Zeiss AG).

### Colony formation assay

Huh7SR and 7721SR cells were seeded in the wells of six-well plates at 1000 cells/well and continuously cultured for 10–14 days. The culture medium was replaced every 3 days. After the colonies had formed, the medium was removed and the cells were fixed with 4% paraformaldehyde for 15 min and then stained with 0.1% crystalline violet. Images were obtained with a camera. Each colony consisted of at least 50 cells.

### Protein half-life assay

Cycloheximide can inhibit the synthesis of new proteins and, therefore, can be used to detect the half-life of protein degradation. In brief, cells were treated with 100 µg/mL of cycloheximide for 0, 5, 30, 60, 90, or 120 min before protein collection. Protein levels were measured by western blot analysis.

### Nuclear and cytoplasmic protein extraction

Nuclear and cytoplasmic proteins were extracted from cultured cells using the Nuclear and Cytoplasmic Protein Extraction kit (P0027; Beyotime Institute of Biotechnology) in accordance with the manufacturer’s instructions.

### The Cancer Genome Atlas (TCGA) and Gene Expression Omnibus (GEO) data analysis

HCC gene expression data and relevant clinical information were retrieved from TCGA and GEO databases. The results are presented as box or violin plots. Genetic correlations were identified with Spearman’s rank correlation test and statistical significance was assessed with the Wilcoxon test.

### Function enrichment analysis

Gene Set Enrichment Analysis (GSEA) enrichment analysis was performed using normalized RNA-Seq data obtained from the TCGA database. Gene ontology terms and pathways in the Kyoto Encyclopedia of Genes and Genomes were assessed to determine the biological functions of FASN. Enrichment results with a false discovery rate (FDR) < 0.25 and adjusted *p* < 0.05 were considered statistically significant.

### Animal experiments

The study protocols involving animals were approved by the Animal Care and Use Committee of the Shanghai Tenth People’s Hospital. Four-week-old male nude mice were purchased from Shanghai Super-B&K Laboratory Animal (Shanghai, China) and subcutaneously injected into the right side with 1 × 10^7^ 7721SR cells. When the volume of the subcutaneous tumors reached approximately 150–250 mm^3^, the mice were randomly assigned to one of four designated groups, which included a negative control (NC) group (equal amount of saline, gavage, 5 days/week), sorafenib group (20 mg/kg, gavage, 5 days/week), orlistat group (240 mg/kg, gavage, 5 days/week), or combination treatment group (20 mg/kg of sorafenib + 240 mg/kg of orlistat, gavage, 5 days/week). The length and width of the tumors were measured every 3 days with calipers, and the tumor volume (V) was calculated as V (mm^3^) = length (mm) × width^2^ (mm^2^)/2. On day 21, the mice were euthanized and the tumors were resected and weighed.

### Statistical analysis

All statistical analyses were performed using GraphPad Prism 8.0 software (GraphPad Software, San Diego, CA, USA). The two-sided unpaired t-test was used to analyze parametric data and the Mann–Whitney U-test for nonparametric data. One-way analysis of variance was performed to assess differences among multiple groups. Spearman’s rank correlation coefficient was employed to identify correlations between the expression levels of different genes. Data are presented as mean ± SD. p-values less than 0.05 were regarded as statistically significant (* p-value < 0.05; ** p-value < 0.01; *** p-value < 0.001).

## Results

### Sorafenib resistance is associated with ferroptosis insensitivity

**Fig. 1:**
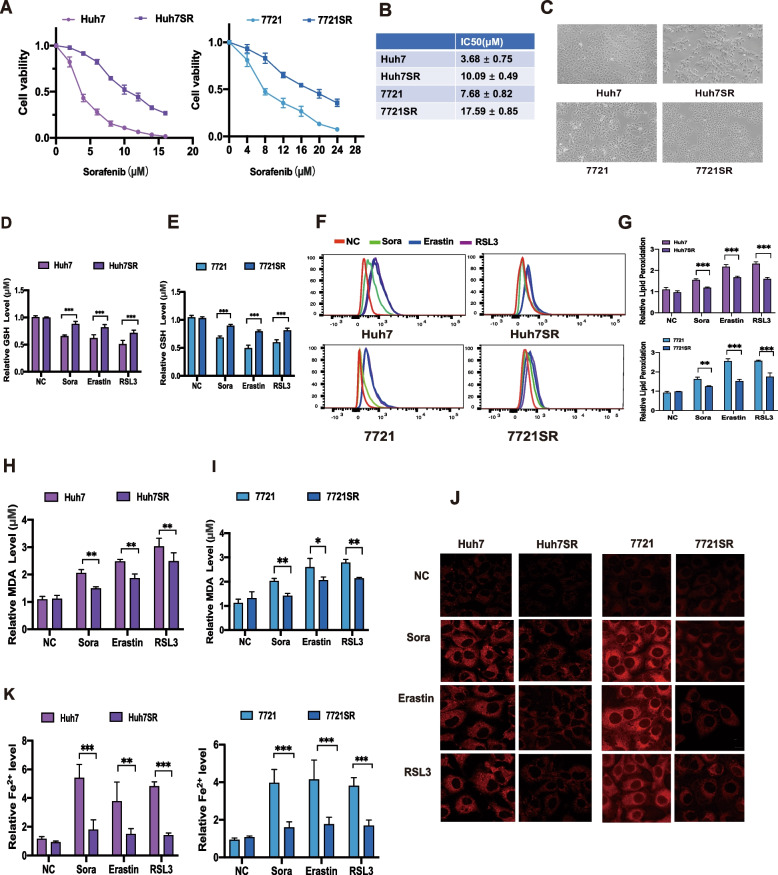
Sorafenib resistance associated with ferroptosis resistance. **A** CCK-8 assay of sensitivity of Huh7, Huh7SR, 7721, and 7721SR cells to sorafenib. **B** 24 h IC_50_ values of Huh7, Huh7SR, 7721, and 7721SR treated with sorafenib. **C** Morphology of Huh7, Huh7SR, 7721, and 7721SR at 200× magnification. **D** Intracellular GSH concentrations of Huh7 and Huh7SR cells exposed to 4 µM sorafenib, 10 µM erastin, and 1 µM RSL3. **E** Intracellular GSH concentrations of 7721 and 7721SR cells exposed to 8 µM sorafenib, 10 µM erastin, and 1 µM RSL3. **F** and **G** Lipid ROS levels of Huh7, Huh7SR, 7721, and 7721SR cells exposed to sorafenib (4 µM for Huh7SR, 8 µM for 7721SR), 10 µM erastin, and 1 µM RSL3. **H** MDA concentrations of Huh7 and Huh7SR cells exposed to 4 µM sorafenib, 10 µM erastin, and 1 µM RSL3. **I** MDA concentrations of 7721 and 7721SR cells exposed to 8 µM sorafenib, 10 µM erastin, and 1 µM RSL3. **J** and **K** Intracellular Fe^2+^ concentrations of Huh7 and Huh7SR cells exposed to 4 µM sorafenib, 10 µM erastin, and 1 µM RSL3 and 7721 and 7721SR cells exposed to 8 µM sorafenib, 10 µM erastin, and 1 µM RSL3. *p < 0.05; **p < 0.01; ***p < 0.001. sora, sorafenib

To investigate the mechanism of sorafenib resistance in HCC, we established two sorafenib resistant cell lines, named Huh7SR and 7721SR, respectively. The half maximal inhibitory concentration (IC50) of sorafenib was significantly higher in Huh7SR and 7721SR cells than the corresponding parental cell lines (10.09 ± 0.49 vs. 3.68 ± 0.75 µM and 17.59 ± 0.85 vs. 7.68 ± 0.82 µM, respectively; Fig. 1A and B). As compared to the parental cells, Huh7SR and 7721SR cells were more elongated with a smaller nucleus-cytoplasm ratio (Fig. 1C). A growing body of evidence indicates that induction of ferroptosis can inhibit tumor growth and overcome drug resistance [6, 20, 21]. Sorafenib, erastin, and RSL3 are representative ferroptosis-inducing compounds that suppress the x_c_^−^ system and expression of glutathione peroxidase 4 (GPX4) [22]. We assessed the sensitivity of Huh7SR and 7721SR to ferroptosis relative to Huh7 and 7721 at the same concentration of inducers and found that the intracellular GSH concentration of Huh7SR and 7721SR cells was significantly higher than that of the corresponding parental cell lines (Fig. 1D and E), while intracellular levels of lipid peroxides, MDA, and Fe^2+^ were lower (Fig. 1F–K). These results suggested that Huh7SR and 7721SR are ferroptosis resistant.

**Fig. 2:**
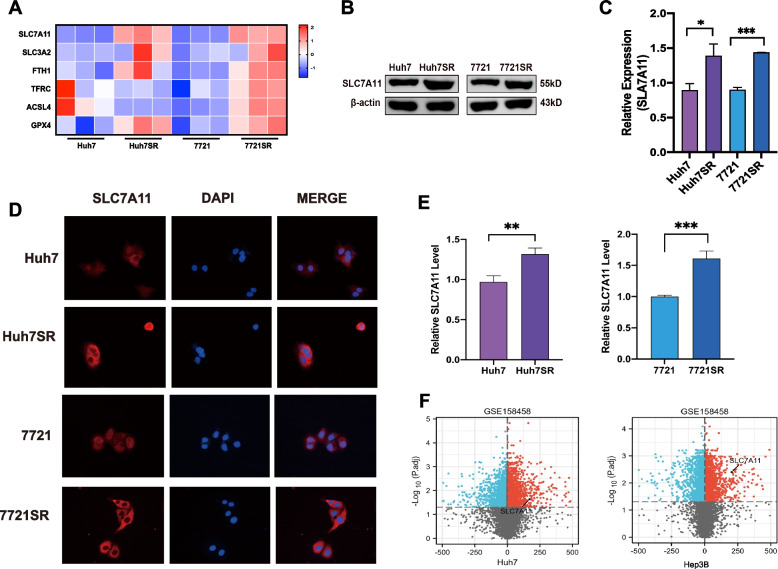
SLC7A11 expression is correlated with sorafenib resistance in HCC cells. **A** Expression of ferroptosis-related genes detected in parental cells and SR cells. **B** and **C** SLC7A11 expression level in parental and SR cells as determined by western blot analysis. **D** and **E** SLC7A11 expression levels in parental and SR cells as determined by IF analysis. **F** Volcano plot showing differentially expressed genes between the parental and resistant cells in the GSE158458 gene set, where SLC7A11 is highly expressed in both sorafenib resistant Huh7 and Hep3B cells. *p < 0.05; **p < 0.01; ***p < 0.001

The expression levels of genes associated with ferroptosis were examined to elucidate the mechanisms underlying ferroptosis resistance in Huh7SR and 7721SR cells. The results showed that SLC7A11 mRNA levels were significantly higher in both Huh7SR and 7721SR cells than the sorafenib-sensitive parental cells (Fig. 2A). Likewise, western blot and IF analyses showed that SLC7A11 protein levels were also significantly higher in both SR cells (Fig. 2B–E). Similar results were found in resistant cell lines established by others, where SLC7A11 expression was significantly increased in sorafenib-resistant cells of both Huh7 and Hep3B cells (GSE158458; Fig. 2F). It indicated that SLC7A11 played an important role in sorafenib resistance of HCC cells.

## FASN upregulation drives acquired resistance to sorafenib

**Fig. 3:**
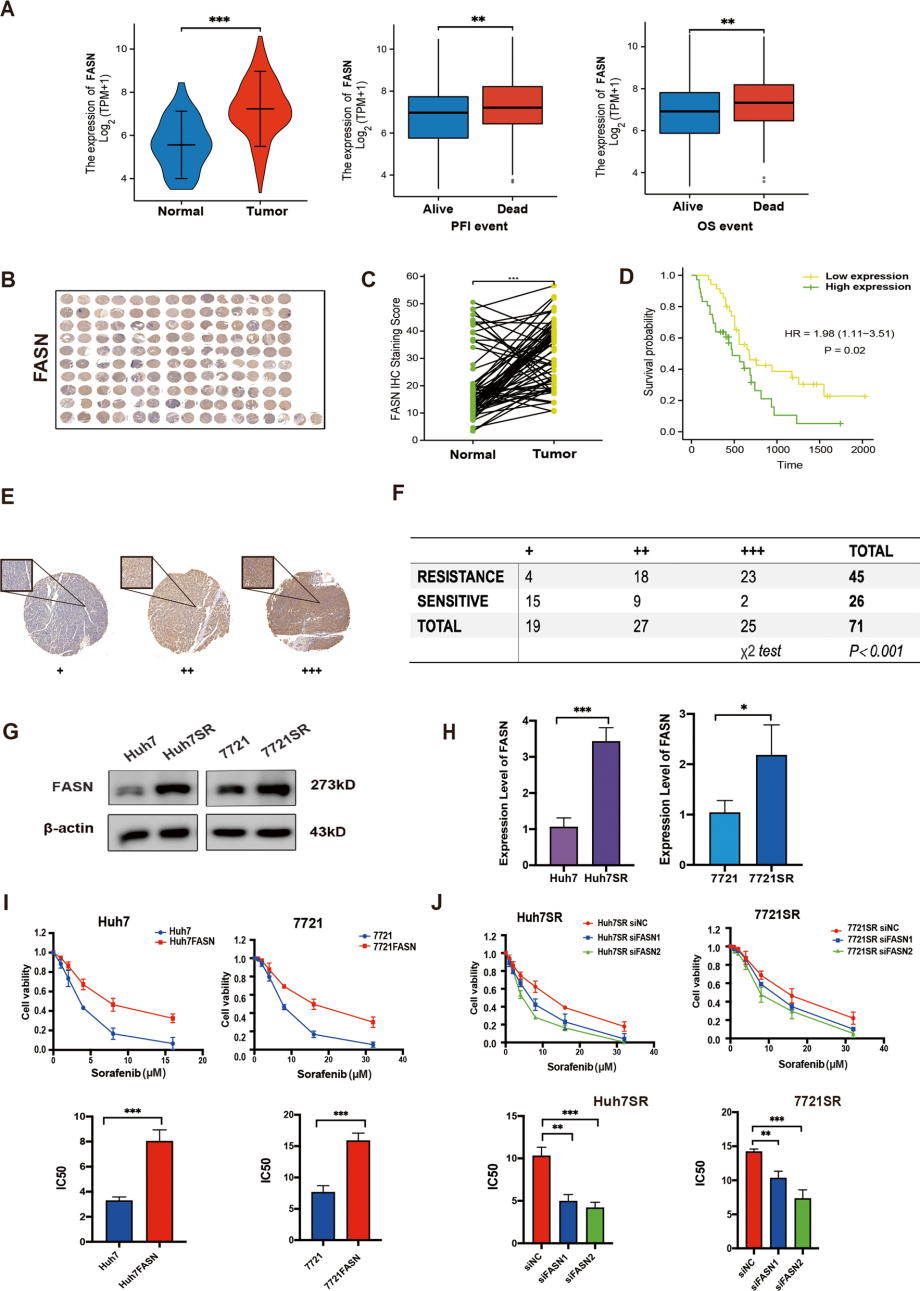
**A** Expression levels of FASN differed between normal and tumor tissues and were correlated with PFI and OS of HCC patients. **B** IHC images of the tissue microarray stained with anti-FASN antibody. **C** IHC staining of FASN in HCC tumor tissue and adjacent tissue according to staining scores of the tissue microarray. **D** Kaplan–Meier survival curves of the correlation between FASN expression levels and overall survival according to the tissue microarray. **E** Enlarged IHC images of FASN staining from the tissue microarray. **F** The data were analyzed using a chi-square test. **G** and **H** FASN protein expression levels in parental and sorafenib-resistant cell lines. **I** CCK-8 assay of sensitivity to sorafenib in FASN-overexpressed Huh7 and 7721 cells versus control Huh7 and 7721 cells and IC_50_ values. **J** CCK-8 assay of the sensitivity to sorafenib of FASN-knock down Huh7SR and 7721SR cells and IC_50_ values. *p < 0.05; **p < 0.01; ***p < 0.001. sora, sorafenib; PFI, progressive-free interval; OS, overall survival; HCC, hepatocellular carcinoma

FASN is the only cytoplasmic enzyme for the *de novo* synthesis of lipids and is reportedly associated with tumor proliferation via resistance to radiation and chemotherapeutic drugs [13, 23, 24]. Further analysis with reference to the TCGA database found that FASN mRNA expression was significantly increased in HCC as compared to the normal control group and there was a strong correlation between FASN mRNA levels and poor progression-free interval (PFI) and overall survival (OS) (*p* < 0.01, Fig. 3A). In vitro, We constructed HCC cell lines characterized with siRNA-mediated down-regulation of FASN and transgene-mediated up-regulation of FASN alone (Fig. S1A and B). CCK8 assays demonstrated that pharmacological inhibition of FASN and siRNA-mediated down-regulation of FASN inhibited proliferation of HCC cells, whereas transgene-mediated up-regulation of FASN had positive effect on HCC cells growth (Fig. S1C). IHC was performed on microarrays from 71 HCC patients and adjacent tissues (Fig. 3B), and the IHC staining scores of FASN of tumor tissue specimens of HCC were significantly higher than those of paracancerous tissue specimens, which were analyzed by paired t-test (Fig. 3C). Moreover, Kaplan–Meier survival analysis also showed poorer OS in the high FASN expression group (*p* = 0.02, Fig. 3D). The poor prognosis of HCC patients is always closely related to drug resistance. Therefore, we performed further analysis of tissue microarrays and found a significant negative correlation between FASN expression levels and sensitivity to sorafenib (Fig. 3E and F). The results of western blot analysis also showed that FASN protein expression was upregulated in both Huh7SR and 7721SR cells (Fig. 3G and H). To further validate the role of FASN in regulation of the response to sorafenib, HCC-SR cells with silenced FASN were established (Fig. S1D). The CCK-8 assay results revealed that overexpression of FASN in Huh7 and 7721 cells significantly inhibited sorafenib-induced cytotoxicity (Fig. 3I), while depletion of FASN resulted in enhanced toxic response to sorafenib in both Huh7SR and 7721SR cells (Fig. 3J). Meanwhile, FASN overexpression promoted the proliferation of Huh7 and 7721 cells when exposed to sorafenib, whereas reduction of FASN expression inhibited the proliferation of Huh7SR and 7721SR cells (Fig. S1E and F). Taken together, these data suggested that FASN up-regulation promoted sorafenib resistance in HCC cells.

### FASN promotes resistance by antagonizing SLC7A11-related ferroptosis

**Fig. 4:**
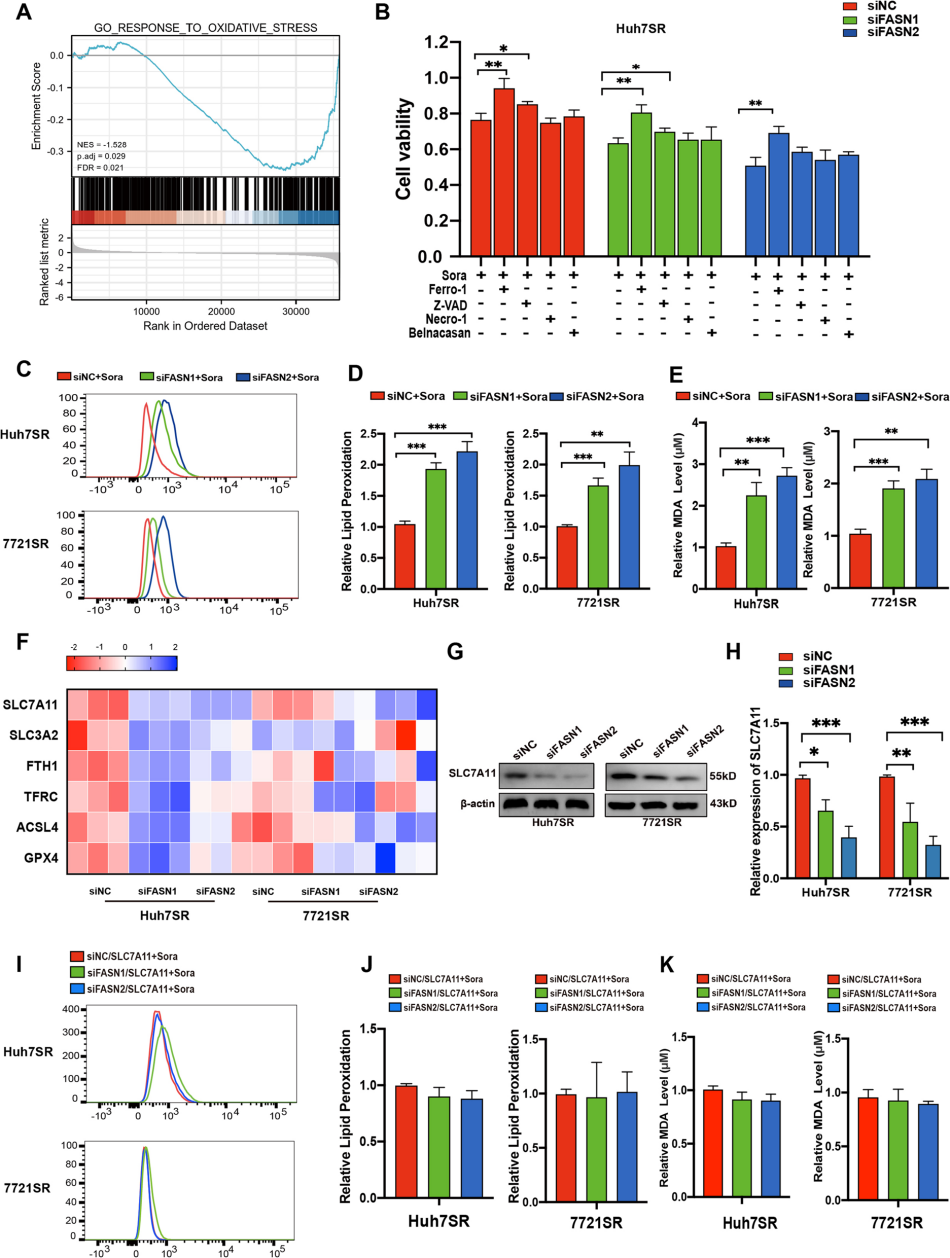
**A** GSEA enrichment analysis of FASN-related genes based on the TCGA database. **B** Proliferation of sorafenib-exposed FASN-knockdown and control Huh7SR cells treated with different cell death inhibitors as determined with the CCK-8 assay. **C** and **D** Detection of lipid ROS levels in FASN-knockdown and control Huh7SR and 7721SR cells exposed to sorafenib as determined by flow cytometry. **E** MDA levels in FASN-knockdown and control Huh7SR and 7721SR cells exposed to sorafenib. **F** Expression levels of ferroptosis-related genes in FASN-knockdown and control Huh7SR and 7721SR cells as determined by RT-qPCR analysis. **G** and **H** SLC7A11 protein expression in FASN-knockdown and control Huh7SR and 7721SR cells. **I** and **J** Effect of SLC7A11 overexpression on lipid peroxidation levels in FASN-knockdown and control Huh7SR and 7721SR cells exposed to sorafenib. **K** Effect of SLC7A11 overexpression on MDA concentrations in FASN-knockdown and control Huh7SR and 7721SR cells exposed to sorafenib. *p < 0.05; **p < 0.01; ***p < 0.001. sora, sorafenib; Z-VAD, Z-VAD-FMK; Ferro1, ferrostatin-1; Necro-1, necrostatin-1

Gene ontology enrichment analysis based on TCGA dataset was performed to further investigate the mechanisms underlying FASN mediation of sorafenib resistance. The results identified significant enrichment of genes associated with the response to oxidative stress (FDR = 0.021, adjusted *p* < 0.029) (Fig. 4A). Ferroptosis is essentially a form of iron-dependent oxidative stress leading to the accumulation of lipid peroxides [25, 26]. Suppression of FASN expression has been found to activate ferroptosis in both Huh7 and 7721 cells with increased levels of intracellular Fe^2+^, lipid ROS, and MDA concentration, while low GSH levels. However, upregulation of FASN inactivated ferroptosis with elevated GSH levels but no effect on levels of intracellular Fe^2+^, lipid ROS, and MDA concentration (Fig. S2A–D). Moreover, blocking the expression of FASN in HCC-SR cells also exhibited elevated lipid peroxidation level (Fig. S2E-F). When FASN-depleted Huh7SR cells and 7721SR cells were exposed to sorafenib, the ferroptosis inhibitor ferrostatin-1, but not the apoptosis inhibitor Z-VAD-FMK, pyroptosis inhibitor belnacasan, or necroptosis inhibitor necrostatin-1, significantly blocked sorafenib-induced cell death (Fig. 4B, Fig. S2G). Moreover, lipid peroxidation and MDA levels were also significantly elevated in FASN-knockdown cells exposed to sorafenib (Fig. 4C–E). These results indicated that blocking of FASN resensitized HCC cells to sorafenib via activation of ferroptosis.

Subsequently, we further explored the potential mechanisms involved in the activation of ferroptosis by blocking FASN. The RT-qPCR results showed that among the genes related to ferroptosis, expression of only SLC7A11 was significantly reduced in FASN-knockdown cells (Fig. 4F). In addition, the results of western blot analysis verified that downregulation of FASN was associated with a notable decrease in SLC7A11 expression in Huh7SR and 7721SR cells (Fig. 4G and H). GSEA enrichment analysis also revealed significant enrichment of FASN-related genes in the REACTOME_SLC_TRANSPORTER_DISORDERS gene set (normalized enrichment score = 2.023; adjusted *p* = 0.046; FDR = 0.036) (Fig. S2H). SLC7A11 is an essential component of the cystine/glutamate retrotransportation system and functions to prevent lipid peroxidation and protect cells from ferroptosis [27]. We overexpressed SLC7A11 in FASN-knockdown Huh7SR and 7721SR cells and it can rescue the lipid peroxidation production and increase MDA concentration when exposed to sorafenib (Fig. 4I–K). Collectively, these results suggested that downregulation of FASN enhanced the response to sorafenib-induced ferroptosis by inhibiting SLC7A11 expression in HCC sorafenib-resistant cells.

### FASN binds to HIF1α to modulate SLC7A11-mediated ferroptosis

**Fig. 5:**
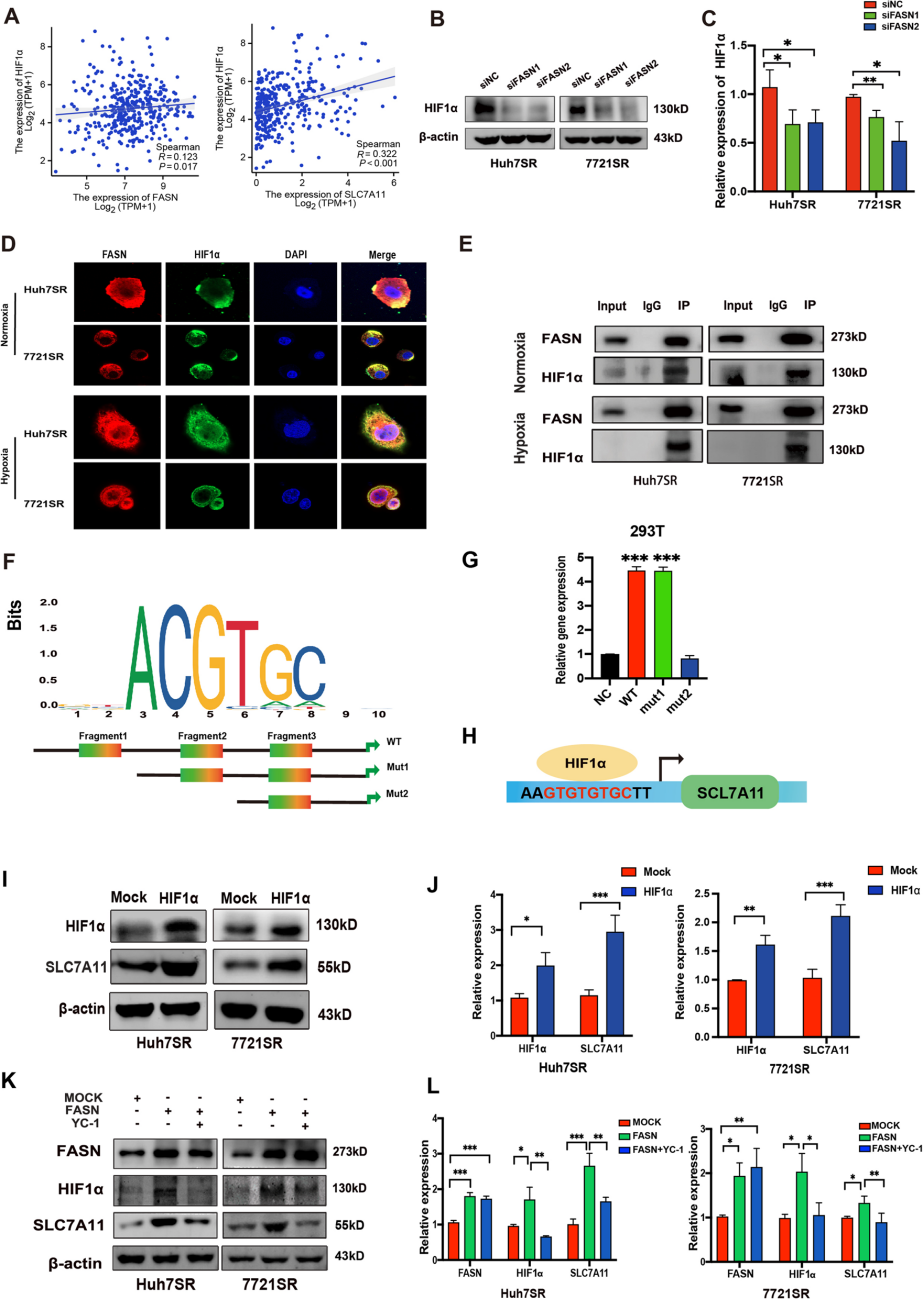
**A** Correlations among HIF1α, FASN, and SLC7A11 based on the TCGA database. **B** and **C** HIF1α protein expression in FASN-knockdown and control Huh7SR and 7721SR cells. **D** Sublocalization of FASN and HIF1α in the cytoplasm in Huh7SR and 7721SR cells under normoxic and hypoxic conditions as determined by IF analysis. **E** Detection of binding between FASN and HIF1α by immunoprecipitation under normoxic and hypoxic conditions. **F** Schematic representation of the HIF1α recognition motif obtained from the JASPAR database (top panel) and three fragments of the SLC7A11 promoter sequence (bottom panel). **G** The pGL4.10- SLC7A11 promoter reporter gene was co-expressed with HIF1α in HEK293T cells for 24 h. Luciferase expression was measured and normalized to *Renilla*. **H** Schematic diagram of HIF1α binding site of the SLC7A11 promoter and the binding sequence. **I** and **J** HIF1α and SLC7A11 protein expression in HIF1α-overexpression and control Huh7SR and 7721SR cells. **K** and **L** Effect of YC-1 on FASN, HIF1α and SLC7A11 protein expression in Huh7SR and 7721SR cells overexpressing FASN. *p < 0.05; **p < 0.01; ***p < 0.001. sora, sorafenib; Z-VAD, Z-VAD-FMK; Ferro1, ferrostatin-1; Necro-1, necrostatin-1

FASN regulation of SLC7A11 expression was further explored. We found that FASN may conjugate to hypoxia-inducible factor-1α (HIF1α) according to BioGRID database (https://thebiogrid.org) and HIF1α coincidentally is an important transcription factor of SLC7A11 based on JASPER database. Meanwhile, bioinformatics analysis based on the TCGA database indicated that HIF1α expression was positively correlated with the expression levels of both FASN and SCL7A11 in HCC (Fig. 5A). HIF1α is a transcription factor that promotes the adaptation of cancer cells to hypoxia conditions, which is an environment most tumors survive in, and overexpression often indicates poor prognosis, drug resistance and metastasis [28, 29]. In Huh7SR and 7721SR cells, silencing of FASN decreased HIF1α expression, while overexpression had an opposite effect (Fig. 5B, C, K), suggesting interactions between FASN and HIF1α. Further, IF and Co-IP analyses confirmed that FASN co-localizes with HIF1α in the cytoplasm under both normoxic and hypoxic conditions (Fig. 5D and E). Moreover, knockdown of FASN significantly increased lipid peroxidation and MDA levels, and HIF1α reversed this effect in sorafenib-exposed HCC-SR cells, indicating that FASN upregulated promoted resistance of HCC-SR cells to sorafenib-mediated ferroptosis via HIF1α (Fig. S3A–B). On the other hand, further experiments demonstrated that supplementation with arachidonic acid (AA) and linoleic acid (LA), essential susceptible PUFAs to lipid peroxidation [30], significantly increased lipid peroxidation and MDA levels in Huh7SR and 7721SR cells (Fig. S3C–D), while accumulated lipid peroxides inhibited the expression of FASN and HIF1α (Fig. S3E). These results suggested that lipid peroxide/FASN/HIF1α can generate positive feedback to amplify lipid peroxidation and ferroptosis.

In addition, the pGL4.10-SLC7A11 promoter reporter gene system was constructed and three luciferase reporter plasmids of different lengths were established based on predicted binding sites (Fig. 5F). The wild-type (WT) SLC7A11 promoter contained fragments 1–3, while truncated mutation 1 (mut1) contained fragments 2 and 3, and truncated mutation 2 (mut2) contained only fragment 3. The WT and mut1 promoters significantly increased luciferase activity in 293T cells, while the mut2 promoter had no significant effect on luciferase activity, indicating that HIF1α binds to fragment 2 of the SLC7A11 promoter (Fig. 5G and H). The results of western blot analysis showed that overexpression of HIF1α indeed increased expression of SLC7A11 and decreased expression of HIF1α, while use of the HIF1α inhibitor YC-1 decreased SLC7A11 expression (Fig. 5I–L). Furthermore, YC-1 reversed upregulation of HIF1α and SLC7A11 induced by overexpression of FASN (Fig. 5K and L). Collectively, these results indicated that FASN regulated SLC7A11 expression by binding to HIF1α in Huh7SR and 7721SR cells.

### FASN promotes nuclear translocation and stabilization of HIF1α

**Fig. 6:**
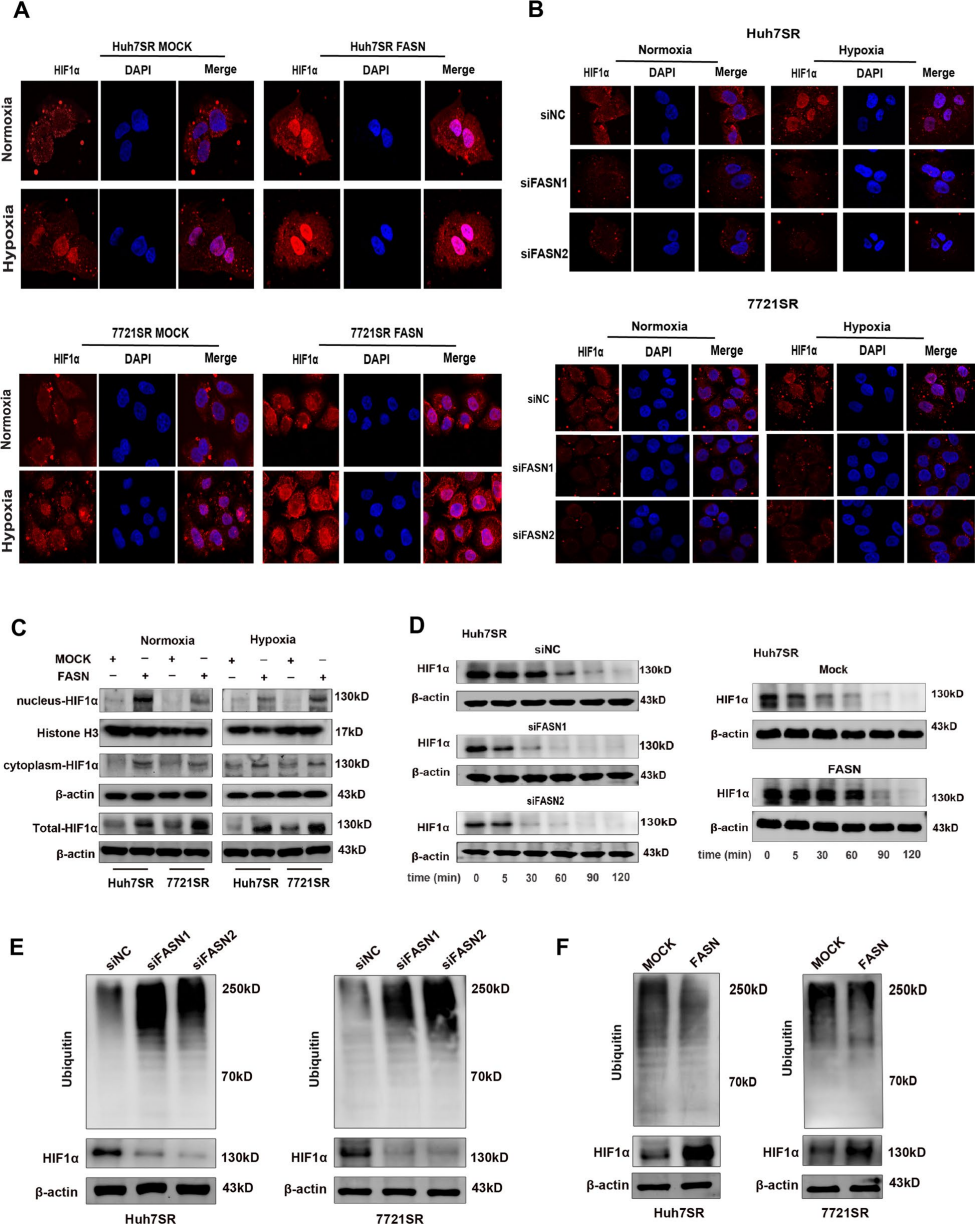
**A** Subcellular localization of HIF1α in Huh7SR and 7721SR cells overexpressing FASN under both normoxic and hypoxic conditions. **B** Subcellular localization of HIF1α in Huh7SR and 7721SR cells depleted of FASN under both normoxic and hypoxic conditions. **C** Nuclear and cytoplasmic expression of HIF1α in Huh7SR and 7721SR cells overexpressing FASN under normoxic and hypoxic conditions. **D** The half-life of HIF1α protein in Huh7SR cells with knockdown and overexpression of FASN. **E** The ubiquitination of HIF1α protein in FASN-knockdown Huh7SR and 7721SR cells. **F** The ubiquitination of HIF1α protein in Huh7SR and 7721SR cells overexpressing FASN

We further characterized the mechanism involved in the regulation of HIF1α by FASN. It has been reported that nuclear translocation of HIF1α plays an essential role in therapeutic resistance [31, 32]. We found that overexpression of FASN promoted the entry of HIF1α into the nucleus either under hypoxic or normoxic conditions in Huh7SR and 7721SR cells, while knockdown of FASN decreased the HIF1α expression in both the nucleus and cytoplasm in Huh7SR and 7721SR cells and there was a significant extranuclearization of HIF1α under hypoxic conditions for Huh7SR and 7721SR cells (Fig. 6A and B). The results of western blot analysis consistently demonstrated that upregulation of FASN promoted nuclear translocation of HIF1α (Fig. 6C). Nuclear translocation can prevent proteins from degradation by several proteases. The results of the cycloheximide assay indicated that overexpression of FASN prolonged the half-life of HIF1α in Huh7SR cells, whereas downregulation had an opposite effect (Fig. 6D). The ubiquitin-proteasome system is the main regulator of HIF1α degradation [33]. Western blot analysis revealed that knockdown of FASN significantly increased ubiquitination of HIF1α in Huh7SR and 7721SR cells, while overexpression of FASN had opposite effects (Fig. 6E and F). These findings suggest that modulation of HIF1α protein levels by FASN was involved in nuclear translocation as well as post-translational modifications.

### Orlistat targeting of FASN resensitized HCC-SR cells to sorafenib both in vitro and in vivo

**Fig. 7:**
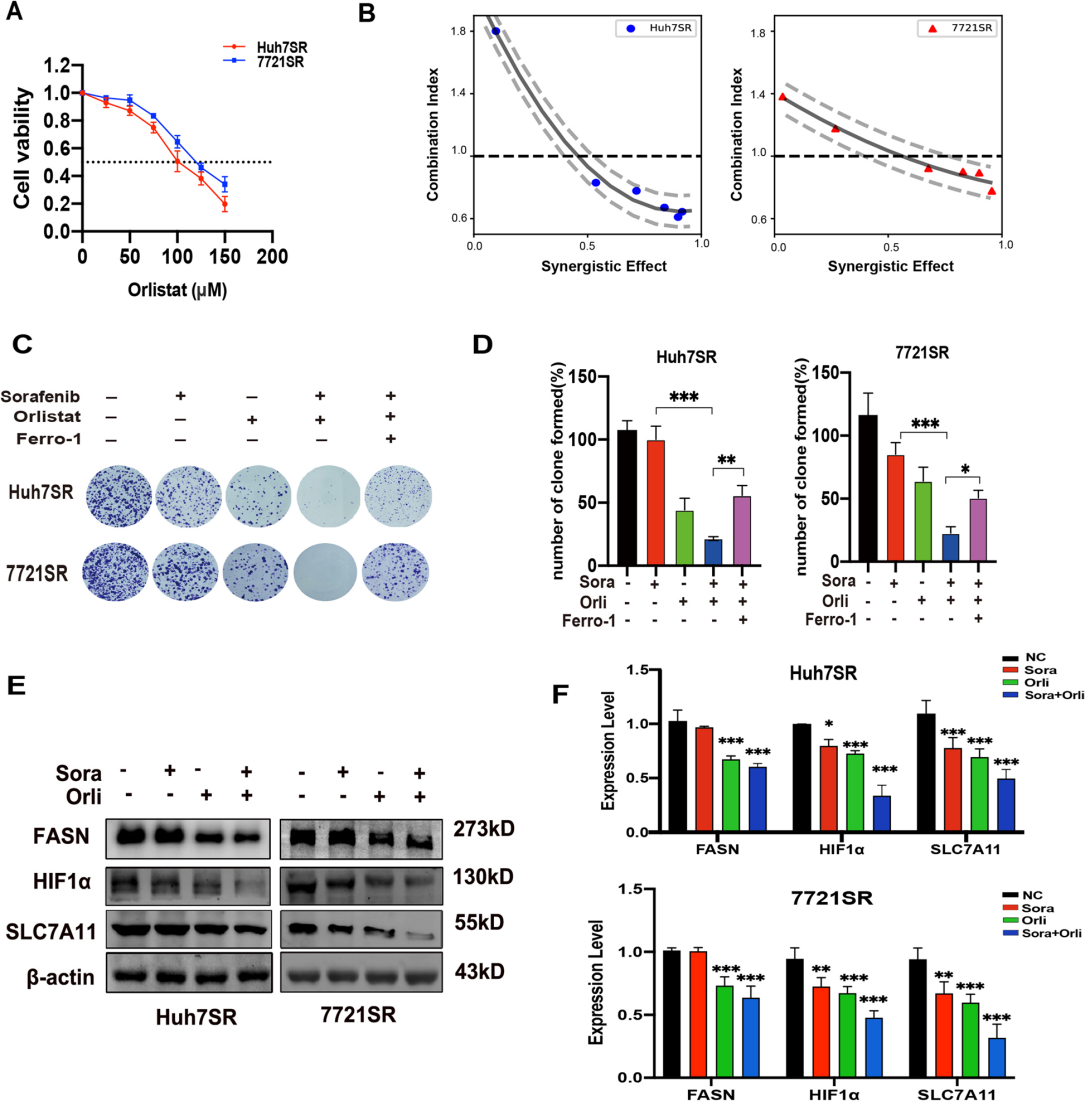
**A** Viability of Huh7SR and 7721SR cells exposed to different concentration of orlistat as determined with the CCK-8 assay. **B** Combination index of sorafenib and orlistat synergistic antitumor calculated with CalcuSyn software. **C** and **D** Antitumor effects of sorafenib combined with orlistat as determined with the colony formation assay. **E** and **F** Alterations to FASN, HIF1α, and SLC7A11 protein expression levels in response to treatment with the combination of sorafenib and orlistat

Orlistat is an inhibitor of FASN and a safe anti-obesity drug approved by the US Food and Drug Administration [34]. The CCK-8 assay was employed to evaluate the inhibitory effect of orlistat against proliferation of Huh7SR and 7721SR cells (IC_50_ = 102.67 ± 6.60 and 133.67 ± 6.56 µM, respectively, Fig. 7A). Then, the co-inhibitory effect of sorafenib and orlistat (1:10) were examined in Huh7SR and 7721SR cells. The combination index was calculated using CalcuSyn software (http://www.biosoft.com/w/calcusyn.htm), where indices of > 1, 1, and < 1 were defined as antagonistic, additive, and synergistic, respectively. The results revealed a synergistic effect of sorafenib and orlistat at relatively low concentrations of 5 µM in Huh7SR cells and 7.5 µM in 7721SR cells (Fig. 7B). The colony formation assay also confirmed the synergistic effect and the obvious proliferation inhibition was partially rescued by the ferroptosis inhibitor (Fig. 7C and D). In addition, the western blot results indicated that orlistat significantly reduced FASN expression, while the combination of sorafenib and orlistat synergistically reduced expression of HIF1α and SLC7A11 in Huh7SR and 7721SR cells (Fig. 7E).

**Fig. 8:**
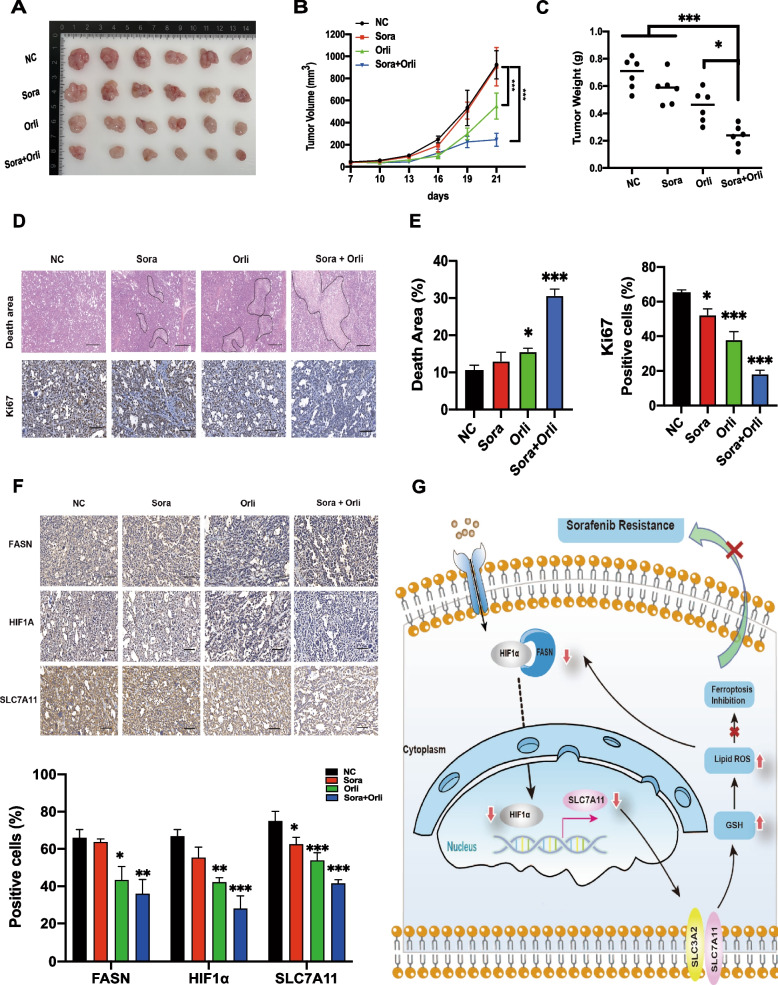
**A** and **B** Volume of subcutaneous tumors in mice of different treatment groups. **C** Tumor weight of subcutaneous tumors in mice of different treatment groups. **D** and **E** Area of death and Ki-67-positive cell ratio of subcutaneous tumors in the four groups of mice. **F** Expression levels of FASN, HIF1α, and SLC7A11 in different treatment groups as determined by IHC analysis. **G** Schematic illustration demonstrating that FASN binding to HIF1α facilitated the entry of HIF1α into the nucleus to promote SLC7A11 transcription thereby inhibiting ferroptosis and promoting sorafenib resistance, while orlistat reversed sorafenib resistance by inhibiting FASN expression. Scale bars, 100 μm. *p < 0.05; **p < 0.01; ***p < 0.001. sora, sorafenib; orli, orlistat; Ferro-1, ferrostatin-1

To evaluate the antitumor effect of the combination of sorafenib and orlistat in vivo, nude mice were subcutaneously injected with 7721SR cells and randomly assigned to one of four groups treated with 20 mg/kg of sorafenib, 240 mg/kg of orlistat, a combination of both drugs (20 mg/kg of sorafenib plus 240 mg/kg of orlistat), or an equal volume of saline by gavage as control group. The results showed that sorafenib had a weak inhibitory effect on drug-resistant tumors, while orlistat significantly retarded tumor growth and reduced the volume and weight and the combination therapy was most effective (Fig. 8A–C). Staining with hematoxylin and eosin and IHC analysis of Ki-67 confirmed that the combination sorafenib and orlistat resulted in extensive tumor cell death and a significant reduction in the proliferation capacity (Fig. 8D and E). In addition, IHC analysis demonstrated that the combination of sorafenib and orlistat inhibited the expression of SLC7A11, the crucial ferroptosis-related protein, through the FASN/HIF1α/SLC7A11 pathway (Fig. 8F). Together, these results suggested that orlistat effectively promoted death of HCC-SR cells via ferroptosis both in vitro and in vivo, thereby resensitizing resistant HCC tumors to sorafenib.

## Discussion

Refractory HCC resulting from drug resistance is a challenge to improve the prognosis of advanced HCC patients. Considering that the metabolism of cancer cells is closely related to drug resistance, new targets associated with lipid metabolism and ferroptosis are needed to ameliorate resistance to sorafenib. The results of this study found that the down-regulation of FASN, an enzyme that regulates *de novo* synthesis of fatty acids, is critical to ameliorate resistance to sorafenib via modulation of ferroptosis. Mechanistically, FASN binds to and upregulates HIF1α, inhibits ubiquitination, and promote nuclear translocation, thereby upregulating the downstream target SLC7A11 and subsequently reducing accumulation of lipid peroxidases, inhibiting HCC ferroptosis, and promoting sorafenib resistance in HCC. Targeting FASN therefore promoted ubiquitinated degradation of HIF1α and inhibited SLC7A11expression, thereby increasing lipid peroxidation in resistant cells and promoting ferroptosis to resensitize HCC cells to sorafenib.

Previous studies suggest that alterations to ferroptosis-related signaling pathways may provide new insights into reversing treatment resistance [35–37]. SLC7A11, a component of the glutamate-cystine antiporter xc^−^, functions to uptake cystine for the synthesis of reduced GSH and subsequently inhibit the accumulation of lipid peroxides [27]. As a key regulator of ferroptosis, overexpressed SLC7A11 is reportedly tightly associated with the efficacy of a variety of chemotherapeutic drugs, targeted drugs, and radiation therapy [37–40]. Since sorafenib is an activator of ferroptosis that targets SLC7A11, it is reasonable to suspect that sorafenib resistance in HCC is closely linked to SLC7A11 expression. The results of the present study found that SLC7A11 expression was most significantly increased in SR cells, which is consistent with a previous report by Huang et al. [6]. Hence, we tried to identify the critical factors involved in the modulation of SLC7A11.

FASN, a pivotal rate-limiting enzyme in fatty acid synthesis, plays a major role in tumor growth and therapeutic resistance, suggesting a potential target in HCC-SR cells. Interestingly, based on the TCGA database, the expression of FASN is closely related to ferroptosis. Indeed, the results of this study demonstrated that FASN modulates sorafenib resistance mainly via ferroptosis. Bartolacci et al. [13] recently reported that FASN depleted activated ferroptosis in lung cancer. In the present study, HCC-SR cells were insensitive to sorafenib-induced ferroptosis when exposed to sorafenib, while depletion of FASN resulted in the accumulation of lipid peroxides, which activated ferroptosis and restored sensitivity to sorafenib. However, the mechanism underlying regulation of ferroptosis by FASN remains unclear. Either based on the results of TCGA-based enrichment analysis or our PCR and western blot results, it was implied that the downstream target of FASN was SLC7A11. Elevated SLC7A11 inhibited accumulation of lipid peroxides induced by depletion of FASN in HCC-SR cells exposed to sorafenib.

The mechanism underlying FASN regulation of SLC7A11 expression in HCC-SR cells was explored based on bioinformatics analysis and previous reports. Gong et al. [41] reported that FASN regulated HIF1α expression in HCC cells, while the results of our study demonstrated for the first time that in HCC-SR cells, FASN bound to and promoted nuclear translocation of HIF1α, while inhibiting ubiquitination and proteasomal degradation, consistent with previous reports of the roles of HIF1α in tumor progression and drug resistance [42–44]. Another recent study demonstrated that sorafenib attenuated fibrosis by inhibiting activation of the HIF1α/SLC7A11 pathway [45] and the same pathway were validated in resistant HCC cells in our experiments. After a comprehensive analysis of the results we obtained through the JASPAR database predictions, we identified the binding site of HIF1α to the SLC7A11 promoter. Overall, the results demonstrated that targeting the FASN/HIF1α/SLC7A11 pathway could significantly promote ferroptosis in HCC-SR cells. In addition, our study also showed that accumulation of lipid peroxides could also inhibit the expression of FASN and HIF1α. Thus, by reducing the expression of FASN can also promote the positive feedback of lipid peroxide/FASN/HIF1α, which further amplifies the lipid peroxidation response and thus further activates ferroptosis in HCC-SR cells.

In parallel, orlistat, as an inhibitor of FASN, exhibited synergistic antitumor effects with sorafenib at low doses. Moreover, we demonstrated that HCC-SR cells can be sensitized to sorafenib-induced ferroptosis both in vivo and in vitro via the FASN/HIF1α/SLC7A11 pathway. Orlistat is clinically used as an anti-obesity drug [46]. Our research offers a promising prospect for future clinical translation.

Overall, our study elucidated the specific and detailed mechanism of how FASN is involved in the modulation of sorafenib resistance in HCC cells. The present study demonstrated that inhibition of FASN expression in combination with sorafenib exhibited a synergistic effect on inhibiting the proliferation of HCC-SR cells. More notably, we also found that orlistat, an inhibitor of FASN, exhibited combination therapeutic efficacy with sorafenib at the indicated concentrations, both in vivo and in vitro, thereby laying a solid foundation for future clinical applications.

## Conclusion

FASN upregulates expression and promotes nuclear translocation of HIF1α, while stabilizing the protein structure and inhibiting ubiquitination-induced degradation, resulting in upregulation of SLC7A11 expression, which can inhibit sorafenib-induced ferroptosis. Moreover, orlistat, the pharmacological inhibitors of FASN, combined with sorafenib exhibited excellent synergistic antitumor effects, both in vivo and in vitro. Our study demonstrated the feasibility of targeting FASN combined with sorafenib as an effective treatment strategy for sorafenib-resistant HCC patients.

## Supplementary Information

**Additional file 1: Table S1.** Primer Sequences. 

**Additional file 2: Supplementary Fig. 1.** (A) Validation of the establishment of Huh7 and 7721 cells with knockdown of FASN. (B) Validation of the establishment of Huh7 and 7721 cells overexpressing FASN. (C) Monitoring the proliferation of Huh7 and 7721 cells in the presence of siRNA-mediated knockdown of FASN, transgene-mediated overexpression of FASN alone, as well as orlistat, a pharmacological inhibitor of FASN with the CCK-8 assay. (D) Validation of the establishment of Huh7SR and 7721SR cells with knockdown of FASN. (E) Monitoring proliferation of FASN-overexpressed and control Huh7 and 7721 cells after exposure to sorafenib (4 µM for Huh7, 8 µM for 7721) with the CCK-8 assay. (F) Monitoring the proliferation of FASN-knockdown and control Huh7SR and 7721SR cells exposed to sorafenib with the CCK-8 assay (4 µM for Huh7, 8 µM for 7721). *p < 0.05; **p < 0.01; ***p < 0.001.

**Additional file 3: Supplementary Fig. 2.** (A) Detection of intracellular Fe^2+^ concentrations in Huh7 and 7721 cells after knockdown of FASN, after overexpression of FASN, and after 24 h incubation with inhibitor orlistat. (B) Detection of intracellular GSH concentrations in Huh7 and 7721 cells after knockdown of FASN, after overexpression of FASN, and after 24 h incubation with inhibitor orlistat. (C) Detection of MDA levels in Huh7 and 7721 cells after knockdown of FASN, after overexpression of FASN, and after 24 h incubation with inhibitor orlistat. (D) Detection of lipid peroxide levels by flow cytometry in Huh7 and 7721 cells after knockdown of FASN, after overexpression of FASN, and after 24 h incubation with inhibitor orlistat. (E) Detection of lipid peroxide levels in FASN-knockdown and control Huh7SR and 7721SR cells by flow cytometry. (F) Detection of MDA concentrations in FASN-knockdown and control Huh7SR and 7721SR cells. (G) Proliferation of sorafenib-exposed FASN-knockdown and control 7721SR cells treated with different cell death inhibitors as determined with the CCK-8 assay. (H) GSEA enrichment analysis of FASN-related genes based on the TCGA database. Scale bars, 10 μm. *p < 0.05; **p < 0.01; ***p < 0.001. sora, sorafenib; Z-VAD, Z-VAD-FMK; Ferro1, ferrostatin-1; Necro-1, necrostatin-1. 

**Additional file 4: Supplementary Fig. 3.** (A) Effect of overexpression of HIF1α on lipid peroxide levels in FASN-knockdown and control Huh7SR and 7721SR cells exposed to sorafenib. (B) Effect of overexpression of HIF1α on MDA concentrations in FASN-knockdown and control Huh7SR and 7721SR cells exposed to sorafenib. (C) Lipid-peroxidation levels in Huh7SR and 7721SR cells after incubated with 2.5 µM AA and 10 µM LA for 24 h. (D) MDA concentrations in Huh7SR and 7721SR cells after incubated with 2.5 µM AA and 10 µM LA for 24 h. (E) Western blot assay showed the expression of FASN and HIF1α in Huh7SR and 7721SR cells after incubated with 2.5 µM AA and 10 µM LA for 24 h. *p < 0.05; **p < 0.01; ***p < 0.001. sora, sorafenib; AA, arachidonic acid; LA, linoleic acid. 

## Data Availability

All the data obtained and/or analyzed during the current study were available from the corresponding authors on reasonable request.
