# Supplementary material for: Targeting fatty acid synthase modulates sensitivity of hepatocellular carcinoma to sorafenib via ferroptosis
Source: J Exp Clin Cancer Res. 2023 Jan 6;42:6. doi: 10.1186/s13046-022-02567-z (PMC9817350; doi:10.1186/s13046-022-02567-z)
Supplement: Supplementary file 1 — Additional file 1: Table S1. Primer Sequences. [file 13046_2022_2567_MOESM1_ESM.pdf]

Table S1. Primer Sequences

	Forward	*Reverse*
SLC7A11	TCTCCAAAGGAGGTTACCTGC	AGACTCCCCTCAGTAAAGTGAC
SLC3A2	CTGGTGCCGTGGTCATAATC	GCTCAGGTAATCGAGACGCC
FTH1	TCCTACGTTTACCTGTCCATGT	GTTTGTGCAGTTCCAGTAGTGA
TFRC	ATCGGTTGGTGCCACTGAATGG	ACAACAGTGGGCTGGCAGAAAC
ACSL4	CATCCCTGGAGCAGATACTCT	TCACTTAGGATTTCCCTGGTCC
GPX4	GAGGCAAGACCGAAGTAAACTAC	CCGAACTGGTTACACGGGAA
β-actin	ATGGATGACGATATCGCTG	ATGAGGTAGTCTGTCAGGT
