# Supplementary figures and images for: Targeting fatty acid synthase modulates sensitivity of hepatocellular carcinoma to sorafenib via ferroptosis
Source: J Exp Clin Cancer Res. 2023 Jan 6;42:6. doi: 10.1186/s13046-022-02567-z (PMC9817350; doi:10.1186/s13046-022-02567-z)

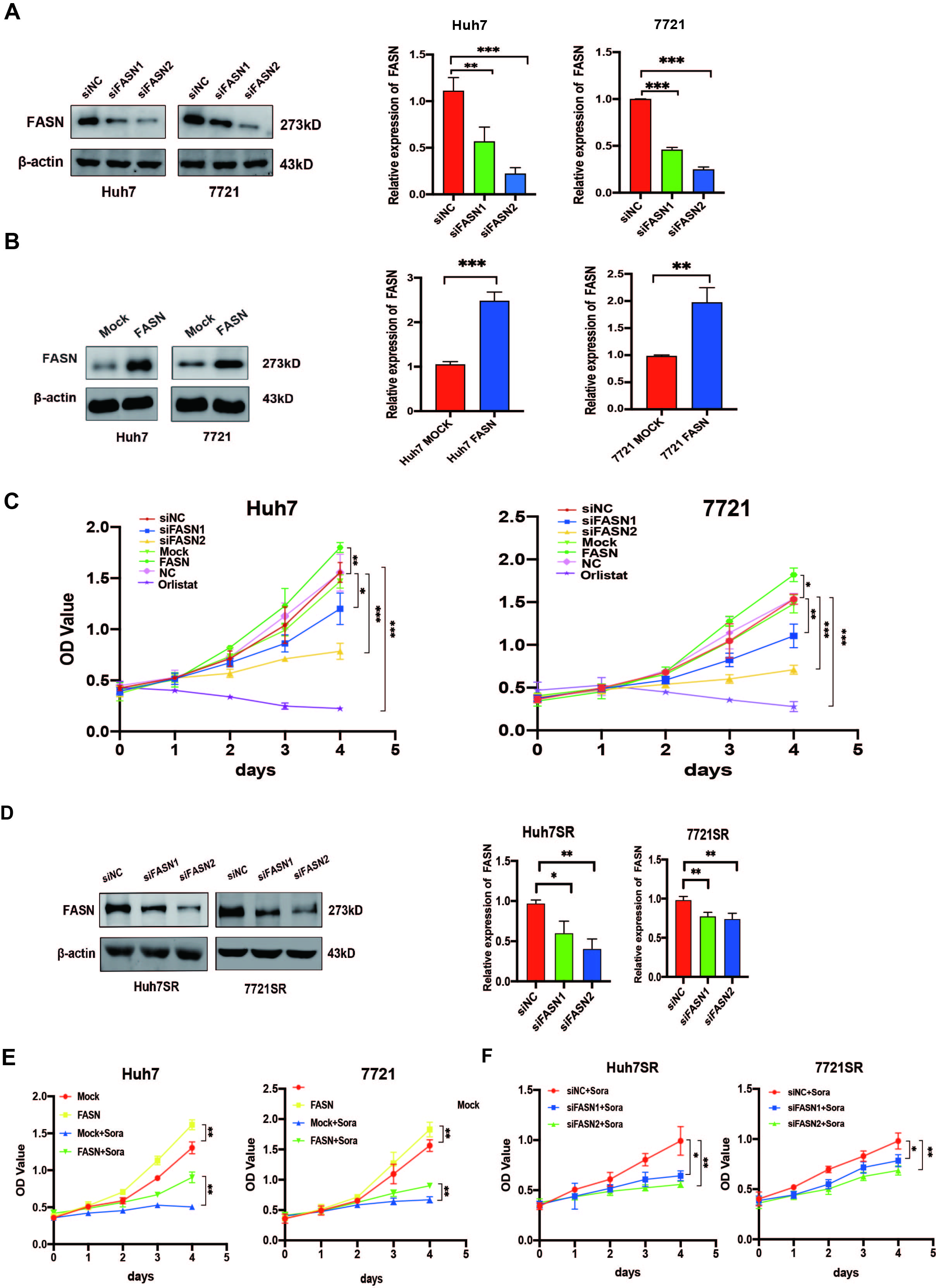

Supplement: Supplementary file 2 — Additional file 2: Supplementary Fig. 1. (A) Validation of the establishment of Huh7 and 7721 cells with knockdown of FASN. (B) Validation of the establishment of Huh7 and 7721 cells overexpressing FASN. (C) Monitoring the proliferation of Huh7 and 7721 cells in the presence of siRNA-mediated knockdown of FASN, transgene-mediated overexpression of FASN alone, as well as orlistat, a pharmacological inhibitor of FASN with the CCK-8 assay. (D) Validation of the establishment of Huh7SR and 7721SR cells with knockdown of FASN. (E) Monitoring proliferation of FASN-overexpressed and control Huh7 and 7721 cells after exposure to sorafenib (4 µM for Huh7, 8 µM for 7721) with the CCK-8 assay. (F) Monitoring the proliferation of FASN-knockdown and control Huh7SR and 7721SR cells exposed to sorafenib with the CCK-8 assay (4 µM for Huh7, 8 µM for 7721). *p < 0.05; **p < 0.01; ***p < 0.001. [file 13046_2022_2567_MOESM2_ESM.jpg]

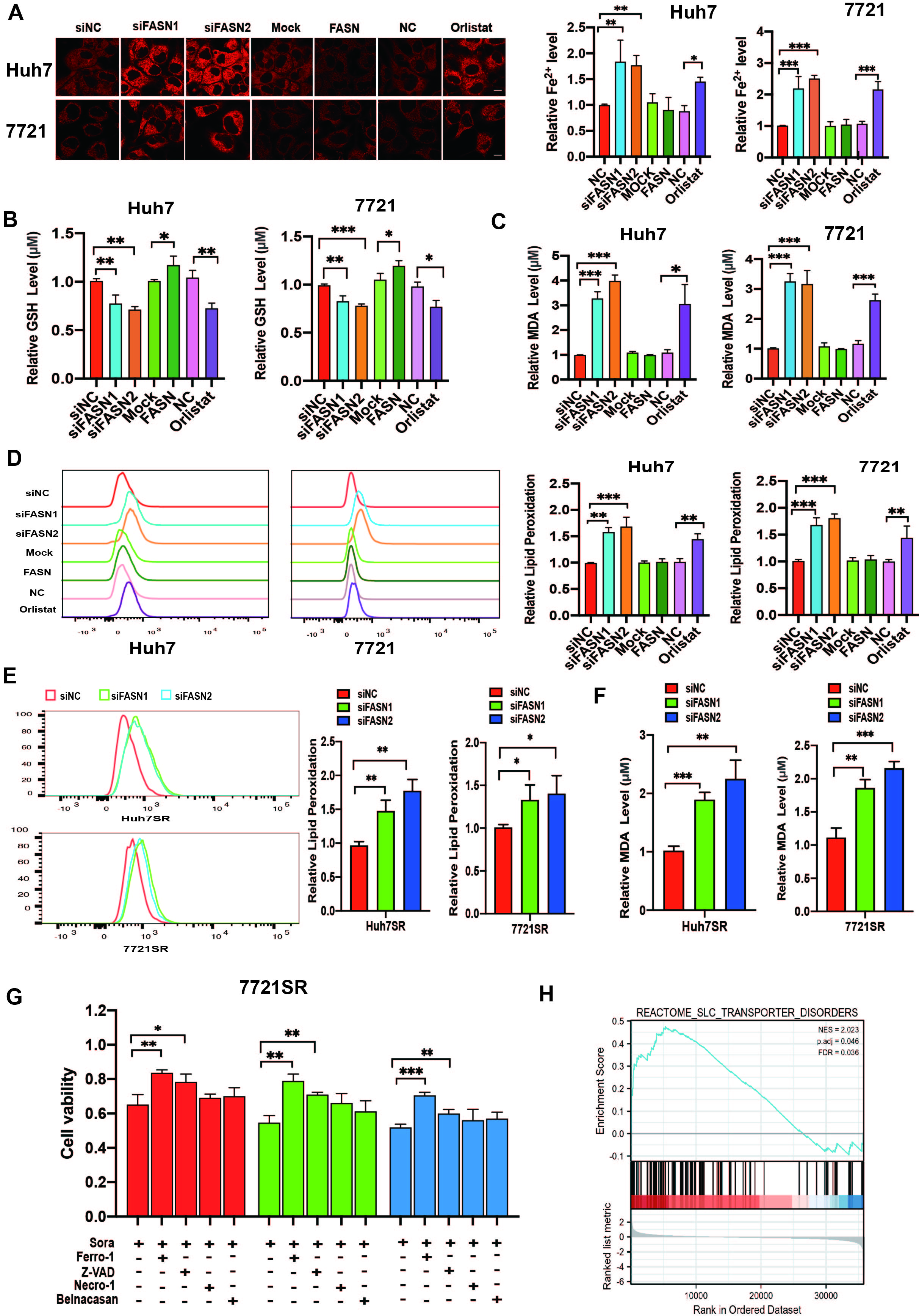

Supplement: Supplementary file 3 — Additional file 3: Supplementary Fig. 2. (A) Detection of intracellular Fe2+ concentrations in Huh7 and 7721 cells after knockdown of FASN, after overexpression of FASN, and after 24 h incubation with inhibitor orlistat. (B) Detection of intracellular GSH concentrations in Huh7 and 7721 cells after knockdown of FASN, after overexpression of FASN, and after 24 h incubation with inhibitor orlistat. (C) Detection of MDA levels in Huh7 and 7721 cells after knockdown of FASN, after overexpression of FASN, and after 24 h incubation with inhibitor orlistat. (D) Detection of lipid peroxide levels by flow cytometry in Huh7 and 7721 cells after knockdown of FASN, after overexpression of FASN, and after 24 h incubation with inhibitor orlistat. (E) Detection of lipid peroxide levels in FASN-knockdown and control Huh7SR and 7721SR cells by flow cytometry. (F) Detection of MDA concentrations in FASN-knockdown and control Huh7SR and 7721SR cells. (G) Proliferation of sorafenib-exposed FASN-knockdown and control 7721SR cells treated with different cell death inhibitors as determined with the CCK-8 assay. (H) GSEA enrichment analysis of FASN-related genes based on the TCGA database. Scale bars, 10 μm. *p < 0.05; **p < 0.01; ***p < 0.001. sora, sorafenib; Z-VAD, Z-VAD-FMK; Ferro1, ferrostatin-1; Necro-1, necrostatin-1. [file 13046_2022_2567_MOESM3_ESM.jpg]

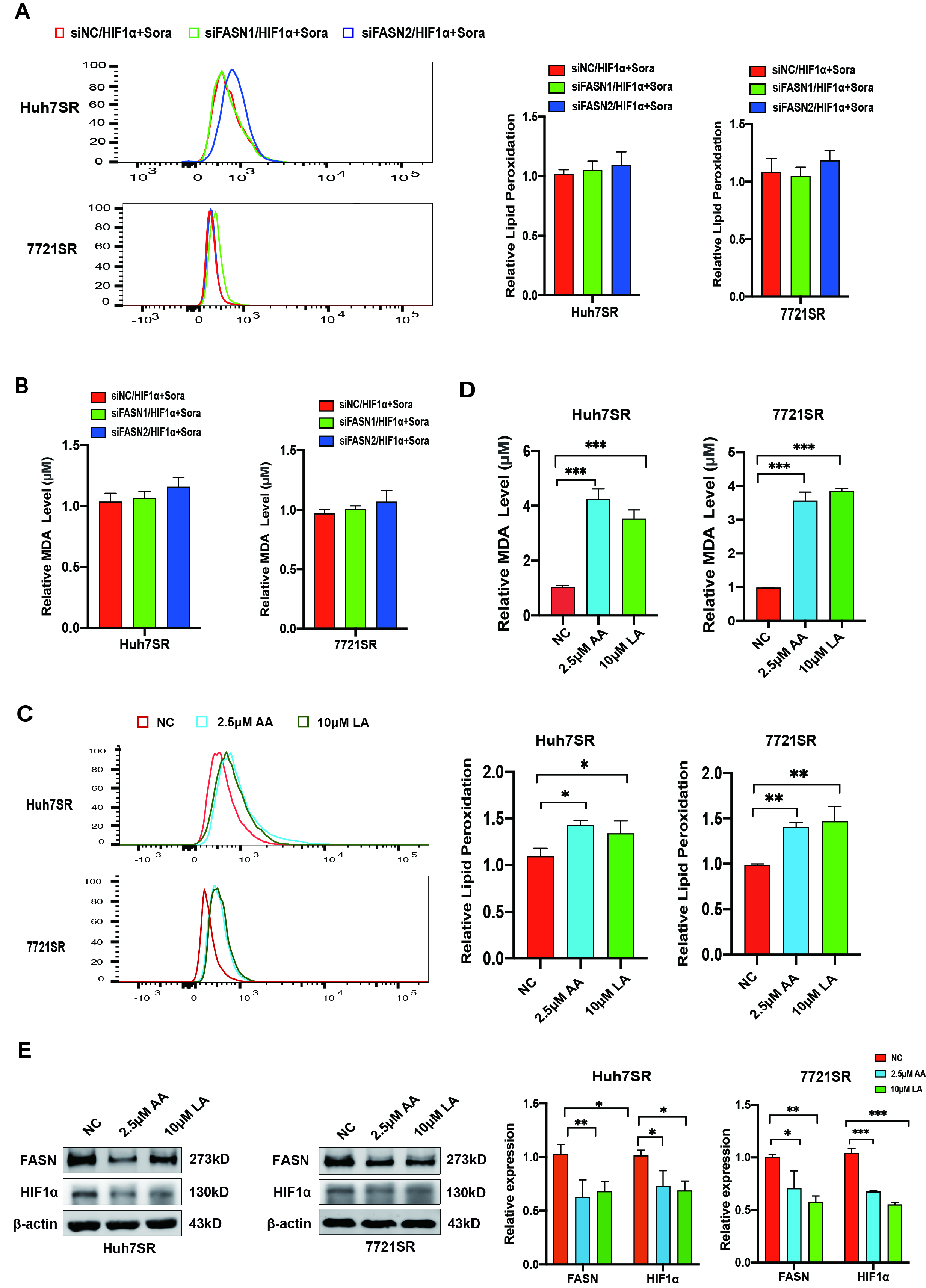

Supplement: Supplementary file 4 — Additional file 4: Supplementary Fig. 3. (A) Effect of overexpression of HIF1α on lipid peroxide levels in FASN-knockdown and control Huh7SR and 7721SR cells exposed to sorafenib. (B) Effect of overexpression of HIF1α on MDA concentrations in FASN-knockdown and control Huh7SR and 7721SR cells exposed to sorafenib. (C) Lipid-peroxidation levels in Huh7SR and 7721SR cells after incubated with 2.5 µM AA and 10 µM LA for 24 h. (D) MDA concentrations in Huh7SR and 7721SR cells after incubated with 2.5 µM AA and 10 µM LA for 24 h. (E) Western blot assay showed the expression of FASN and HIF1α in Huh7SR and 7721SR cells after incubated with 2.5 µM AA and 10 µM LA for 24 h. *p < 0.05; **p < 0.01; ***p < 0.001. sora, sorafenib; AA, arachidonic acid; LA, linoleic acid. [file 13046_2022_2567_MOESM4_ESM.jpg]
